# Effect of Different Enriched Vermicomposts, Humic Acid Extract and Indole-3-Acetic Acid Amendments on the Growth of *Brassica napus*

**DOI:** 10.3390/plants11020227

**Published:** 2022-01-16

**Authors:** Arash Hemati, Hossein Ali Alikhani, Ladan Ajdanian, Mehdi Babaei, Behnam Asgari Lajayer, Eric D. van Hullebusch

**Affiliations:** 1 Department of Soil Science, Faculty of Agriculture, University of Tabriz, Tabriz 5166616422, Iran; h-asgari@tabrizu.ac.ir; 2 Department of Soil Science, University College of Agriculture and Natural Resources, University of Tehran, Tehran 1417466191, Iran; halikhan@ut.ac.ir; 3 Department of Horticultural Sciences, Faculty of Agriculture, Ferdowsi University of Mashhad, Mashhad 9177948974, Iran; ladan137214@yahoo.com (L.A.); mehdi.babaei11@ut.ac.ir (M.B.); 4 Institut de Physique du Globe de Paris, Université de Paris, CNRS, F-75005 Paris, France

**Keywords:** growth stimulant, rapeseed, plant growth performance, bacteria, vermicompost

## Abstract

Humic acid (HA) is a specific and stable component of humus materials that behaves similarly to growth stimulants, esp. auxin hormones, contributing to improving growth indices and performance of plants. As a rich source of HA, vermicompost (VC) is also a plant growth stimulating bio-fertilizer that can enhance growth indices and performance in plants. The purpose of the present study is to compare the influence of VC enriched with bacterial and/or fertilizer, commercial humic acid (CHA) extract, and indole-3-acetic acid (IAA) on improving growth characteristics and performance of rapeseed under greenhouse conditions. The results showed the complete superiority of VC over the CHA and IAA (approximately 8% increase in the dry weights of root and aerial organ and nearly three times increase in seed weight). The highest values of these indices were obtained with VC enriched with Nitrogen, Sulfur, and Phosphorus, *Azotobacter chroococcum* and *Pseudomonas fluorescens*; the lowest value was obtained with VC enriched with urea. Additionally, the application of 3% VC and the control involved the highest and lowest values in all traits, respectively. The SPAD (chlorophyll index) value and stem diameter were not significantly affected by different application levels of VC. Overall, the applications of IAA and the CHA were not found to be suitable and therefore not recommended.

## 1. Introduction

Oil seeds are the second largest source of nutrients after cereals. In addition to being rich in fatty acids, these products contain proteins as well [1]. Rapeseed is known as one of the most important oil seed plants in the world; accordingly, it is considered as the third largest source of vegetable oil after soybean and palm [2].

Vermicompost (VC) is a plant growth stimulating organic fertilizer being a rich source of humic acid (HA), that can improve plant growth performance indices [3]. Vermicompost contains enzymes and natural growth stimulants that, along with nutrients and HA, promote plant growth and yield [4]. The HA obtained from VC is capable of competing with its commercial counterpart that are often produced from coal or leonardite, and even indole phytohormones (esp. IAA) [5]. Several studies have been conducted regarding the effect of VC application on the growth and development of crops [6,7,8]. For instance, for Maize (*Zea mays*), it has been shown that VC increased plant growth yield by creating favorable conditions for plant nutrition [9]. It has also been reported that VC usually contains more nutrients than plant-derived organic matter, and large amounts of these nutrients have been converted into forms that are easily absorbed by plants [10]. On the other hand, VC has high microbial and enzymatic activity and contains large amounts of plant growth regulators [11]. Continuous and adequate use of VC along with proper management can increase organic carbon storage and retain water in soils. Moreover, by improving VC physical properties, it can have a beneficial effect on the growth and yield of plant species [9]. It has also been observed that by increasing the amount of VC in the culture medium, the amount of elements such as zinc, calcium, and nitrogen significantly increase in the plant aerial parts [12]. Furthermore, with the application of VC in field conditions, an increase in barley (*Hordeum vulgare*) yield was observed [13]. Vermicompost treatment in chickpea (*Cicer arietinum*) and pea (*Pisum sativum*) significantly increased morphological traits such as root length, stem length, and the number of leaves [14,15,16]. It was also reported that the application of VC enriched with other nutrients compared to their separate application was accompanied by improved morphological and physiological traits of the corn plant (*Zea mays*) [17].

One of the practical drawbacks of VC is its high-volume consumption (at least 2 to 5 tons per hectare). To solve this problem, in some studies, the population change of microorganisms in VC and the improvement of the quality of organic fertilizers have been tested recently [18]. In addition to nitrogen fixation, some nitrogen-fixing bacteria also dissolve insoluble mineral phosphates by producing organic acids [19,20]. A number of diazotrophic bacteria such as *Pseudomonas*, *Burkholderia*, *Agrobacterium*, *Azotobacter* and *Ervinia* are able to increase absorbable phosphorus and bio-stabilize nitrogen. Increasing the bioavailability of phosphorus by these microorganisms is achieved by producing organic acids that increase the amount of absorbed mineral phosphorus [21]. *Thiobacillus*-inoculated VC has positive effects on the conversion of phosphate soil to absorbable phosphorus, and *Burkholderia* and *Herbaspirillum* genus strains are also reported to increase absorbable phosphate [22,23]. Inoculation of nitrogen-fixing microorganisms increases the amount of nitrogen in VC and inoculation of phosphate-solubilizing microorganisms in VC with the presence of phosphate soil and even without phosphate soil increased phosphorus in VC. However, the direct application of phosphate soil was not particularly useful in natural soils [24,25,26]. Enrichment of compost with ammonium sulfate and urea by adding nitrogen in solid or soluble forms at the beginning of composting process has increased the total nitrogen and increased efficiency in the field of plant growth [27,28].

Humic acid is one of another best plant growth stimulant. This substance is formed by the decomposition of organic matters, especially the plant-derived ones, and is found in soil, coal and peat. Humic acid that is a mixture of very large molecules with the ability to complex metallic elements, is one of the most important components of humus [29]. Consuming HA improves cation exchange capacity and aeration by creating a layer on soil particles and it increases root growth [30]. The effect of HA on spring wheat (*Triticum aestivum*) yield has been investigated by Dinçsoy et al. [31]. The results showed that HA increased the access to phosphorus and other nutrients and also caused a significant yield increment. Experiments carried out on different plants have displayed that HA increases plant growth directly and indirectly, and in different amounts for various plants [30,32]. The direct and positive effect of HA on the growth of wheat [33], chickpeas (*Cicer arietinum*) [34] and chicory (*Cichorium intybus*) [35] have been reported. The plant growth response curve related to HA treatment showed that with increasing HA concentration, plant growth increased [36]. This stimulatory effect at low concentrations could be more related to the direct effect on the plants, which was the effect of natural hormones, along with the indirect effect on the metabolism of soil microorganisms, the dynamics of nutrient uptake from the soil, and the physical condition of the soil [8]. The most important and abundant auxin in plants is IAA, which in general, depending on its concentration, can have different effects on plant growth [37]. The evaluation of the effect of IAA (2000, 1000 and 3000 mg L^−1^) on the improvement of *Balanites aegyptiaca* growth for 24 h, showed a significant increase in the height, number of branches and leaves, chlorophyll content and dry weight of the plant [38].

Considering the importance of rapeseed in oil production and the international societies’ approach to the conservation of natural resources in line with reducing the use of chemical fertilizers, the present study was designed and implemented on the application of bio-fertilizers including VC as a rich source of HA. To this end, different vermicomposts (VCs) enriched with nitrogen, NSP (Nitrogen, Sulfur, Phosphorus) chemical elements, and *Pseudomonas fluorescens* and *Azotobacter chroococcum* at various levels were compared with the commercial humic acid (CHA) and the IAA hormone (as substances that are naturally present in VC). The use of enriched VC aims at reducing the amount of organic materials to be spread on agricultural lands. On the other hand, the quasi-hormonal properties of some compounds contained in VC and its HA content which are expected to stimulate plant growth requires the comparison of different amendment combinations (hormonal substances and HA to be compared with VC amendments enriched with different chemical and biological agents) needs to be investigated. Such an approach has not been tested before and, therefore, represents the novel aspect in this research.

## 2. Materials and Methods

### 2.1. VC Production and Selecting Enrichment Treatments

Vermicompost was produced from cow manure raw materials and plant residues in a 1:3 ratio (weight: weight) in the presence of composting worms (*Eisenia fetida*) for a period of 5 months in the VC Education and Research Station of Agriculture and Natural Resources Campus in Tehran University. To this aim, first cow manure and plant residues were placed under sunshine for 1 month; then, small dome-shaped hills with a width of 70, length of 200, and height of 50 cm were formed. Following sufficient irrigation monitored by the occurrence of leachate, *Eisenia fetida* worms were inserted into the bed (500 earthworms per 100 kg of bed). The humidity of the hills was approximately 50–60% during the composting period, and the humidity was maintained through daily irrigation. By the end of the processing period, earthworms were separated from the final product (VC). The characteristics of the VC were analyzed and are presented in Table 1 [39]. In order to enrich VC with bacterial treatments, phosphate solubilizing bacteria (*Pseudomonas*) and nitrogen-fixating bacteria (*Azotobacter*) were used. *Azotobacter* and *Pseudomonas* belong to the *Azotobacter chroococcum* and *Pseudomonas fluorescens* species, respectively. The entire used strains were obtained from the beneficial terricolous microorganism gene bank of the department of soil science engineering at Tehran University which have been identified, separated, and maintained in studies conducted in previous years [40]. After the second bacterial enrichment, the inoculated bacterial population was adjusted at 4 × 10^9^ cfu (Colony Forming Unit) mL^−1^ and 25 mL of each liquid enrichment was used for inoculation per 1 kg of VC [23]. In addition, it was attempted for this study to make use of necessary chemical elements with the highest degrees of importance and consumption in agriculture (nitrogen, phosphorus, and sulfur) as fertilizer treatments to enrich VC. One percent of each element was added to VC; the source of added nitrogen was urea, the source of phosphorus was triple superphosphate and the source of sulfur was elemental sulfur. Potassium was not added due to the richness of potassium in the produced VC. Prior to enrichment, VC samples were screened using a 2 mm sieve and their large particles were separated.

### 2.2. Soil Amendment Composition and Application Level

Factors investigated in this experiment included 4 VC fertilizer treatments at 4 fertilizing levels including indole-3-acetic acid (IAA) was provided by the University of Tehran (Forbes Pharmaceuticals company, Maharashtra, India); 75% pure CHA from Leonardite imported from China (Hebei China Company, Hebei, China) was obtained from the market. Vermicompost was completely mixed with the soil at 4 levels of 0, 1, 2, and 3% (equivalents to 0, 30, 60 and 90 g pot^−1^). Additionally, the CHA at 4 levels of 0, 200, 400, and 600 mg/kg and IAA acid with concentrations of 10^−6^, 10^−5^, and 10^−4^ molar, 100 mL per pot (Table 2) were used in three 10, 20, and 30 day stages as well as two flowering and reproductive stages through hilling. The concentrations used to study was selected based on previous data published in the literature for CHA [5,41,42,43] and IAA [44,45]. The use of HA and IAA were both accumulated around the crowns of plants and the added VC was completely mixed with pot soil.

### 2.3. Plant Growth and Material

Pots were placed in the greenhouse environment after being prepared. The growing stage was carried out by manually placing five shrubs in plastic pots containing 3 kg of soil from 20 April to 22 September 2012. The loamy soil used in this study was prepared from the research farm of Karaj Soil and Water Institute located in Meshkin Dasht, Karaj. Sampling was performed from a depth of 0–30 cm of the soil surface and the samples were transferred to the laboratory. Some physicochemical properties of the soil sample were measured after air drying, crushing, and passing through a 2 mm sieve, and are presented in Table 3. Overall, the measurements of available P was done by the Olsen method, total nitrogen was done by the Kjeldahl method, available K by using 1 N acetate ammonium, available Zn and Fe estimated by DTPA-TEA extraction method, EC and pH in saturated soil extract, and soil texture by the hydrometric method [46,47]. Generally, this experiment was conducted based on analysis of soil used in this study. Malakouti et al. [48] reported that the critical levels of N, P and K varies between 1000 and 2000, 25 and 450 and 8 and 15 mg kg^−1^ soil, respectively, based on soil characteristics. Since the soil used in the present study showed a deficiency in N and P, the amendments used in the experiments were enriched in N, P and/or bacterial strains; phosphate solubilizing and nitrogen-fixing bacteria (*Pseudomonas fluorescens* and *Azotobacter chroococcum*, respectively).

After drying, the soil was passed through a 4 mm sieve for use in pots. After germination and complete settlement of plant germs, their numbers were narrowed down to two per pot. In this study, the modified RGS (spring cultivar and sensitive to cold) rapeseed cultivar was used which was obtained from Karaj Seed and Seedling Research Institute. The minimum and maximum temperatures of the greenhouse was 20 and 28 °C, respectively, with a relative humidity of 75–80%. In addition, the rapeseed shrubs were exposed to 14 h of light (a combination of fluorescent and tungsten lamps), daily.

The plants growth period was completed within four months during which the pots were visited daily, and the humidity of each pot were adjusted at 0.75–0.8 field capacity in terms of weight. The harvesting stage began by the end of the growth period, after the plants grew clusters. After being separated from the soil and weighing the wet weight of aerial organs, these organs were washed entirely using distilled water; next, they were placed inside clean paper envelopes and then dried in an oven for 48 h at 65 °C; subsequently, the dry weight of the aerial organ was measured as well. Then, the dried aerial organ was separately powdered using a grinder and then placed inside lidded containers to produce herbal extracts and perform analytical experiments. Furthermore, the root system of the plant was completely taken out of the soil as much as possible and then placed in a basin full of water; next, the surrounding soil was washed away and ultimately, the wet and dry weights of the root was measured.

### 2.4. Measuring Plant Characteristics after Harvesting

The height of the shrub was measured from crown to the tip of the stamen in centimeters, without taking the root into account. The root was carefully separated from the soil and washed with water to remove its surrounding soil as much as possible. After separating the root from the soil, its length was measured in centimeters using a ruler. All leaves for each shrub was counted during the growing and harvesting periods. The length of the largest inflorescence in each shrub was measured in centimeters from the stem growth location. The length of the largest inflorescence was solely measured due to difference in the number of inflorescences under various treatments; moreover, measuring the lengths of all inflorescences and indicating inflorescence average length would not have represented the reality of the study in a few cases. Stem diameter was measured in millimeters using calipers from the stem, under the first knuckle. To determine the extent of chlorophyll in a leaf, SPAD-502 manual chlorophyll meter (Minolta, Japan) was used without damaging plant textures and extracting from leaves. To this end, three leaves were selected on average during the flowering stage and the extent of chlorophyll was estimated from its central point. Leaf area was measured using a leaf area meter tool, model Delta T-Devices UK (**ΔT** Area Meter MK2). The extent of photosynthesis was measured in µM CO_2_ × m^−2^ × s^−1^. Stomatal conductance was measured via an aerial infrared gas analyzer (IRGA) tool, model LCA4-ADC. At the end of the 4-month period and after the clusters were formed and dried, the number of clusters, percentage of fertile clusters and the total seed weight were measured for each plant according to Equation (1).

Fertile Clusters (%) = (Number of Fertile Clusters/Total Number of Clusters) × 100 (1)

#### 2.4.1. Evaluating the Amount of Oil Contents in Rapeseed

The oil existing inside the seeds was extracted using Soxhlet method, via methanol-chloroform organic solvent in a 1:2 ratios and three repetitions. The method was used on rapeseed for the first time by Joshi et al. [49]. The dried seeds were powdered, transferred to M_3_ Whatman Filter Paper, and weighed (Weight A). The sample was then packed tightly and placed inside an oven for 6–8 h and was dried until reaching a stable weight (Weight B). After cooling at room temperature in a desiccator, samples were transferred to Soxhlet pipes and were extracted for 24 h via petroleum ether (b.p. below 50 °C). Following extraction, the packed samples were placed under a hood to evaporate the remaining petroleum ether and become dried; ultimately, desiccator was cooled and then weighed (Weight C). Oil contents (%) was calculated according to Equation (2).

Oil content (%) = (B − C)/(B − A) × 100%(2)

The dried seeds were triturated and transferred to 3M Whatman filter paper and weighed (weight A); the sample was tightly closed and then dried in an oven for 6–8 h until a stable weight was reached (weight B). After cooling to room temperature in a desiccator, the samples were transferred to Soxhlet tubes and extracted with petroleum ether for 24 h (b.p. below 50 °C). Then, after extracting the packaged samples for evaporation, the remaining petroleum ether was placed in the hood to dry and finally, the desiccator was cooled and then weighed (weight C). Three repetitions were prepared for each sample and the average value of these three treatments was employed to calculate the amount of oil contents.

#### 2.4.2. Ashing Plant Materials and Producing Herbal Extraction

The dry ashing method was employed to produce herbal extract. To this end, 1 g of ground dry plant material was poured into a crucible and then placed inside a furnace; temperature was gradually raised to 450 °C so that white ash is produced. After the samples were cooled, 20 mL of 2 normal hydrochloric acids was added to each sample and then placed in a sand bath for 30 min. Finally, samples were filtered in 100 mL volumetric flask and brought to volume [50].

#### 2.4.3. Total Phosphorus Analysis

In order to measure phosphorus, the yellow method (Molybdovanadate) was employed. Accordingly, the plant sample solution was prepared following the preparation of phosphorus yellow and standard solutions. First, 20 mL of the herbal extract produced using the dry ashing method was poured into a 100 mL volumetric flask; then, 20 mL of yellow indicator and 20 mL of distilled water were added. After 45 min, the solution was brought to volume and phosphorus content was read using a spectrophotometer at 430 nm wavelength. Prior to reading plant samples, the standard solutions were read using the device and its chart was obtained [50].

#### 2.4.4. Measuring Copper Concentration in Plant’s Aerial Organ

The concentration of copper in herbal extracts was measured and reported using an atomic absorption spectroscopy (Shimadzu AA-670). For this assay, 0.1 g of the dried plant organs of each pot were digested with 2 mL of 60% nitric acid overnight and then placed in a water bath for 2 h at 90 °C. After cooling, 1 mL of hydrogen peroxide was added to the samples and the tubes were placed in a water bath at 90 °C for half an hour. After cooling, the samples were reduced to 10 mL with distilled water. Copper extracted from the root and aerial parts of the plant were measured using a device during Shimadzu AA-670 atomic absorption spectrometry and a calibration curve was drawn with respect to the exclusive wavelength of each element; finally, samples were read [50].

### 2.5. Experiment Design and Statistical Analysis

Greenhouse experiments were performed as a factorial in the form of randomized complete block design (RCBD) (with 2 factors including treatment and treatment level) in four repetitions. The obtained results were analyzed using SAS software and the related variance analysis tables were drawn. Additionally, the comparison of data mean values was performed by using Duncan’s multiple range test at 5% level via MSTAT-C software.

## 3. Results and Discussion

### 3.1. Effects of Amendment Formulation on Morpho-physiological Characteristics of Rapeseed

#### 3.1.1. Plant Height, Stem Diameter, and Number of Leaves

**Figure 1:**
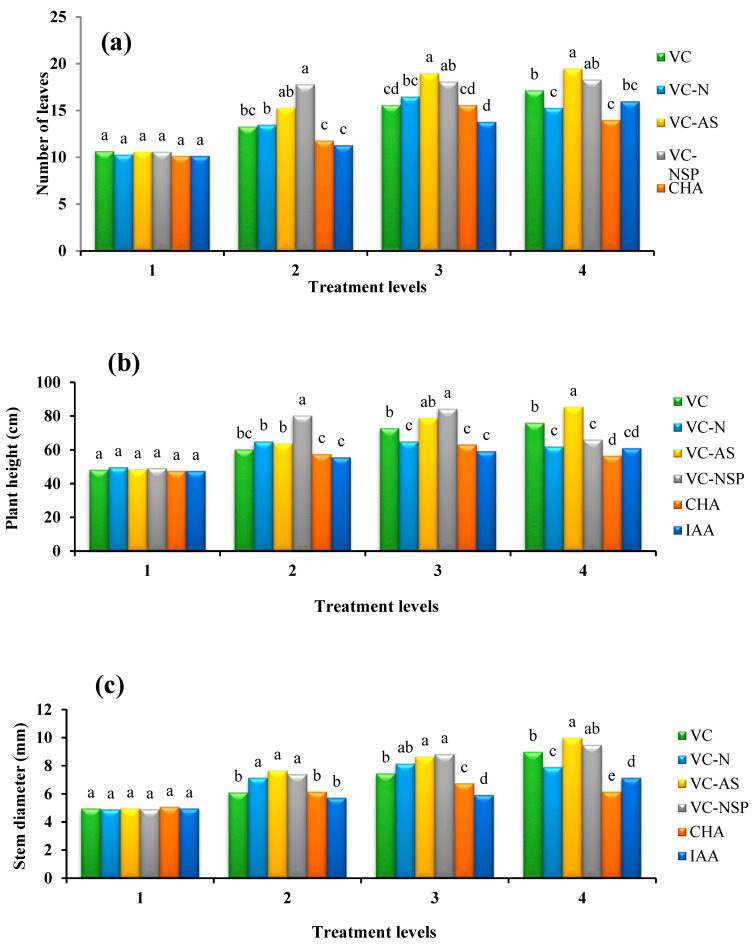
Comparison of average soil application of experimental treatments on number of leaves (**a**), plant height (**b**) and stem diameter (**c**) at treatment levels (levels 1, 2, 3 and 4 for VC, VC-N, VC-AS and VC-NSP treatments of 0, 1, 2 and 3% VC, 0, 200, 400 and 600 mg of CHA per kg of soil and 0, 10^−6^, 10^−5^ and 10^−4^ molar IAA). Different letters in each figure show significant difference at *p ≤* 0.05 by Duncan multiple range test. VC, VC without enrichment; VC-N, VC enriched with 1% nitrogen; VC-NSP, VC enriched with 1% nitrogen, 1% sulfur, and 1% phosphorus; VC-AS, VC enriched with *Azotobacter chroococcum* (21Az) + *Pseudomonas fluorescens* (Ps 59); CHA, Commercial humic acid; IAA, Indole-3-acetic acid.

The effect of the applied soil amendments on the above traits was significant at 1% probability level (*p ≤* 0.01). As indicated in Figure 1a, the highest number of leaves was observed in VC-AS treatment at 4% application, which was significantly different from the control and VC-N treatment. At similar levels, plant height was the highest in VC-AS treatment, which was significantly different from all VC treatments used at this level (Figure 1b). Additionally, the highest amount of stem diameter was observed in the same treatment used at the 4% level, which was different from other treatments (Figure 1c).

The positive effects of VC enrichment with bacteria have been well identified [51,52]. For instance, while investigating the effect of inoculating VC with bacteria such as *Azotobacter chroococcum* and *Pseudomonas fluorescens*, it was reported that inoculating of VC with these bacteria significantly increased phosphorus and nitrogen in VC relative to the control, which consequently lead to increased nutrient uptake and plant growth indices [53,54]. On the other hand, it has been described that composting with the microbial community dissolves insoluble phosphates and thus increases the available phosphorus content [55]. Some nitrogen-fixing bacteria, in addition to nitrogen fixing, dissolve phosphorus by producing organic acids, which leads to increased plant growth [22]. Based on Figure 1, there was no significant difference in leaf number and stem diameter between VC-AS and VC-NSP treatments. In this regard, it could be concluded that both treatments could increase the amount of nitrogen and phosphorus uptake by plants. However, compared to the control treatment, which is non-enriched VC, a significant difference was observed. Additionally, the results showed that the lowest number of leaves, height and stem diameter was observed in the IAA treatment at the level of 2%. Compared to other concentrations of CHA used, the application of 400 mg/kg CHA leads to the highest values in height and number of leaves in rapeseed. The use of IAA phytohormone at 10^−4^ molar level involved the highest values between all concentrations that used for IAA as well.

El-Nemr et al. [56] reported similar results. Since plant growth is substantially dependent on soil fertility parameters, it appears that the improved physical, chemical, and biological characteristics of the cultivation bed with applied VC are the reasons behind the improved plant growth rate compared to other treatments [57]. Additionally, the relative advantage of VC can be attributed to increased plant growth due to growth stimulants such as plant growth hormones, humic materials, microbial activity and biodiversity, and improved soil fertility [5,58]. Research conducted to assess the effects of VC on the studied plants and its comparison with the effect of CHA as well as HA mixed with plant growth hormones and IAA suggest that VC had the best performance on the experimented plants among other treatments. It was also observed that increased application of VC would increase medical plant (*Moringa oleifera*) root growth and the number of fruits [59].

#### 3.1.2. Leaf Area and SPAD Index

**Figure 2:**
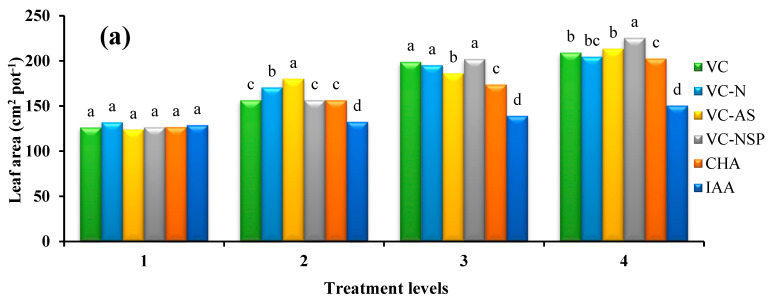

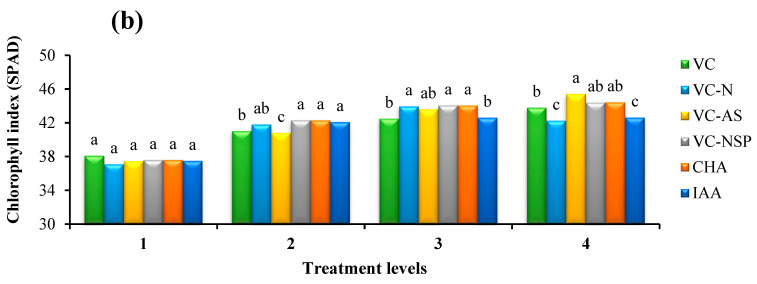
Comparison of average soil application of experimental treatments on leaf area (**a**) and chlorophyll index (SPAD) (**b**) at treatment levels (levels 1, 2, 3 and 4 for VC, VC-N, VC-AS and VC-NSP treatments of 0, 1, 2 and 3% VC, 0, 200, 400 and 600 mg of CHA per kg of soil and 0, 10^−6^, 10^−5^ and 10^−4^ molar IAA). Different letters in each figure show significant difference at *p ≤* 0.05 by Duncan multiple range test. VC, VC without enrichment; VC-N, VC enriched with 1% nitrogen; VC-NSP, VC enriched with 1% nitrogen, 1% sulfur, and 1% phosphorus; VC-AS, VC enriched with *Azotobacter chroococcum* (21Az) + *Pseudomonas fluorescens* (Ps 59); CHA, Commercial humic acid; IAA, Indole-3-acetic acid.

Results obtained from variance analysis of leaf area traits and SPAD index demonstrated a significant difference between various treatment levels. The results of comparing mean value with Duncan’s multiple range test at 5% probability level showed that VC had a higher performance than CHA and IAA; notably, among VC treatments, 3% levels VC-AS and VC-NSP treatments led to larger leaf area and higher SPAD index, respectively. The largest leaf area with a value of 225.5 cm^2^ per pot was observed under 3% level of VC-NSP. The CHA involved a higher value of leaf area index compared to IAA acid soil application treatment with 34.64% increase (Figure 2a). Increasing the concentration and consumption percentage or treatments increased the overall SPAD index; the highest SPAD index value was observed as 46.43 cm^2^ per pot with VC-AS at 3% level. The lowest values for these indices were observed at 0% level treatments (Figure 2b). Plant absorption and assimilation are, to a considerable extent, controlled by the two main factors of leaf area and photosynthesis per leaf area unit. Accordingly, increasing these two factors would enhance plant growth and performance [60].

Improvements in plant growth conditions have been shown with treated enriched VC application [51,52]. Mahanta et al. [61] reported that the chlorophyll content of leaves and the activity of some enzymes in rice were significantly increased under the treatment of VC enriched with *Azotobacter* and *Azospirillum*. Additionally, an increase in shoot growth and yield of cowpea (*Vigna unguiculata*) has been reported following the addition of enriched VC, and this increase was ascribed to the enhanced availability of nutrients of enriched VC, which in turn increases leaf chlorophyll content [62]. However, in our study, we did not observe a significant difference in chlorophyll content (highest level used) between the enriched treatments. It can be concluded that enrichment with bacteria and three important nutrients such as N, P, and S were both able to increase the nutrients availability to plants and, therefore, there was no significant difference between them.

Numerous studies suggest that several complex compounds are formed between humic materials and mineral ions during the humification process; this, in turn, increases enzyme stimulation, their impact on raising respiration intensity, photosynthesis, and nucleic acid metabolism. Additionally, quasi-hormonal activity of humic materials was also reported [63,64,65]. Asciutto et al. [66] also reported increased leaf area in *Impatiens walleranaas* a result of 100–75% VC treatment application. Berova and Karanatsidis [67] observed increased photosynthetic pigments in pepper leaves following the application of VC. Golchin et al. [68] reported that the leaf area index and chlorophyll contents of pistachio leaves were higher under VC treatment compared to other treatments without VC. The highest rates were observed in 10% and 20% VC treatments, which increased by about 60%. Nutritional elements used for chlorophyll production such as nitrogen, phosphorus, potassium, manganese, iron, and copper are easily accessible to plants under VC treatment [69].

#### 3.1.3. Stomatal Conductance and Photosynthesis

**Figure 3:**
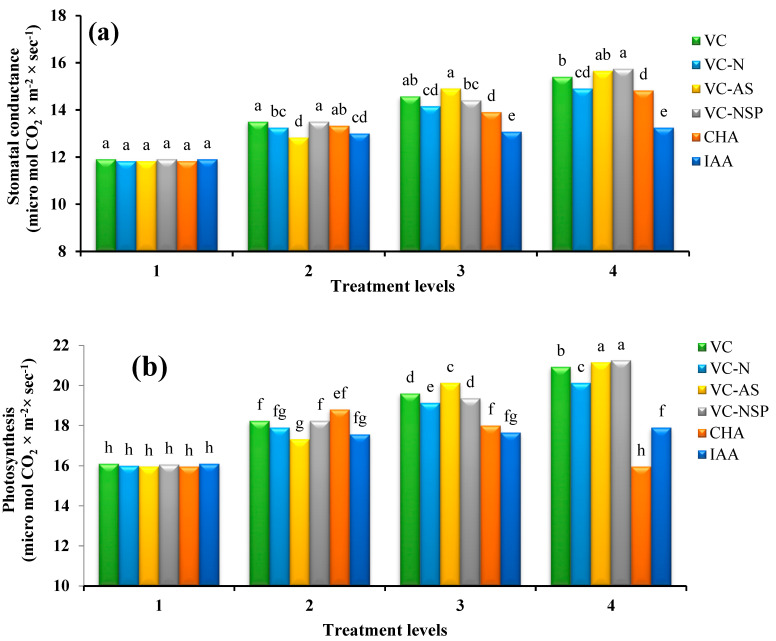
Comparison of average soil application of experimental treatments on stomatal conductance (**a**) and photosynthesis (**b**) at treatment levels (levels 1, 2, 3 and 4 for VC, VC-N, VC-AS and VC-NSP treatments of 0, 1, 2 and 3% VC, 0, 200, 400 and 600 mg of CHA per kg of soil and 0, 10^−6^, 10^−5^ and 10^−4^ molar IAA). Different letters in each figure show significant difference at *p ≤* 0.05 by Duncan multiple range test. VC, VC without enrichment; VC-N, VC enriched with 1% nitrogen; VC-NSP, VC enriched with 1% nitrogen, 1% sulfur, and 1% phosphorus; VC-AS, VC enriched with *Azotobacter chroococcum* (21Az) + *Pseudomonas fluorescens* (Ps 59); CHA, Commercial humic acid; IAA, Indole-3-acetic acid.

The results of mean value comparisons showed significant difference between various treatments. In addition, VC-AS and VC-NSP treatments involved higher stomatal conductance and photosynthesis while IAA treatment yielded the lowest values of both indices. The highest extents of stomatal conductance and photosynthesis were observed under the 3% VC-NSP treatment with values of 15.57 and 21.26 µM CO_2_ × m^−2^ × s^−1^, respectively (Figure 3a,b). Increasing the concentration and consumption percentage of treatments raised the overall values of both indices. Photosynthesis in plants is influenced by both internal and external factors as well as environmental conditions. The internal factors of a given cultivar determine the potential photosynthesis capacity. One of the internal factors affecting the extent of photosynthesis is the health of leaves and presence of sufficient chlorophyll within them; succulence of leaves results in increased photosynthesis rates [70]. A variety of factors including accessibility to sufficient water supply, suitable humidity conditions, absence of water stress, and high water potential of leaves affect the extent of photosynthesis, directly or indirectly [71]. There is a direct relation between stomatal conductance and photosynthesis in which increasing the former would enhance the latter. Parallel changes between photosynthesis and stomatal conductance demonstrate the fact that maintaining photosynthesis can be attributed to retaining stomatal conductance [72].

### 3.2. Effect of Using Different Treatments on Dry and Wet Weights of Aerial Organ, Root, and Aerial Organ to Root Ratio

**Figure 4:**
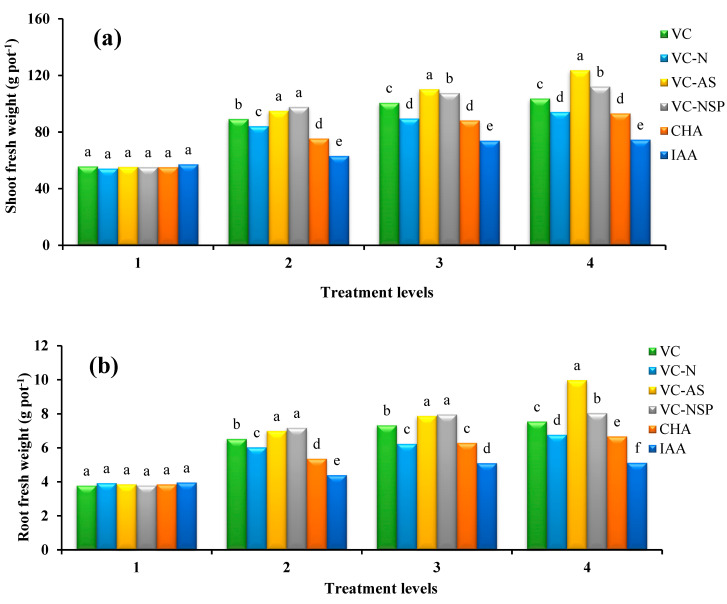

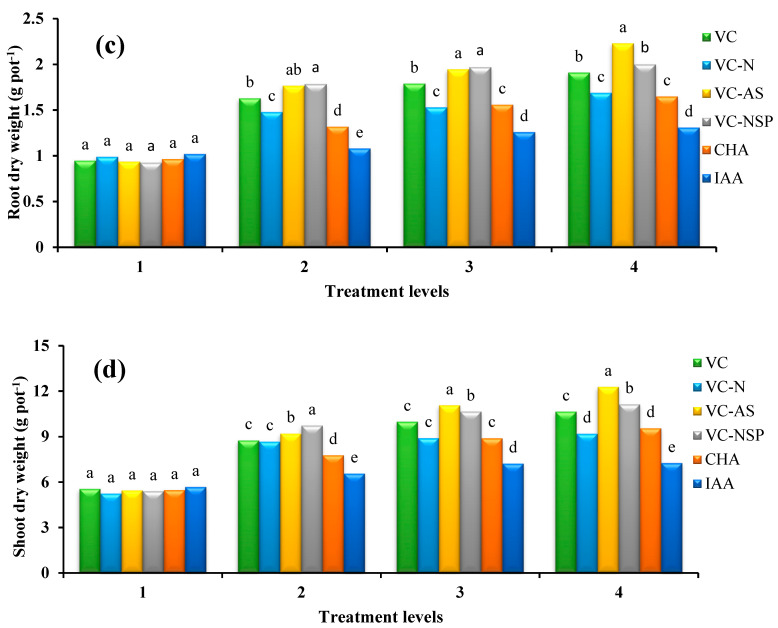
Comparison of average soil application of experimental treatments on shoot fresh weight (**a**), Root fresh weight (**b**), root dry weight (**c**) and shoot dry weight (**d**) at treatment levels (levels 1, 2, 3 and 4 for VC, VC-N, VC-AS and VC-NSP treatments of 0, 1, 2 and 3% VC, 0, 200, 400 and 600 mg of CHA per kg of soil and 0, 10^−6^, 10^−5^ and 10^−4^ molar IAA). Different letters in each figure show significant difference at *p ≤* 0.05 by Duncan multiple range test. VC, VC without enrichment; VC-N, VC enriched with 1% nitrogen; VC-NSP, VC enriched with 1% nitrogen, 1% sulfur, and 1% phosphorus; VC-AS, VC enriched with *Azotobacter chroococcum* (21Az) + *Pseudomonas fluorescens* (Ps 59); CHA, Commercial humic acid; IAA, Indole-3-acetic acid.

The effect of different amendments applied at various quantity levels were found to be significant at 1% probability level for the entire set of said traits. The results of the mean value comparison showed that the highest values for wet and dry weights of the aerial organ and root were obtained from applying the 3% VC treatment. The highest wet and dry weight values of the aerial organ were 123.7 and 12.28 g, respectively (Figure 4a,d); the wet and dry weights of the root were also found to be 9.98 and 2 g, respectively (Figure 4b,c). The lowest and highest values for the dry weights of aerial organ to root ratio were obtained from the applications of IAA and VC-AS and VC-NSP treatments, respectively. A positive correlation was observed between shoot and root dry weight with increasing concentration of treatments used. Asciutto et al. [66] observed that increasing the amount of VC (75–100%) at the cultivation bed would enhance the dry weights of both the aerial organ and root in *Impatiens walleranaas*. The use of VC positively affects dry matter, seed performance, protein contents, and plants’ nutrient absorption. Such a positive impact is probably due to the higher amounts of nutritional elements which, in turn, results in the availability of macro and micro nutrients [73]. Chanda et al. [57] reported enhanced performance as a result of increased VC amounts. Such an increase is speculated to be the result of higher amounts of accessible nitrogen which is necessary for the production of structural proteins. Compost and VC contain large amounts of humic materials (About 5 to 15 percent have been reported in various studies) in addition to nutrients and organic materials; by enhancing the bioavailability of particular nutrients, esp. iron and zinc [74] and directly affecting plant metabolism [75] these materials increase plant growth and performance.

### 3.3. Effect of Using Different Treatments on Seed Performance, Inflorescence Lengths, and Oil Percentage in Seeds

**Figure 5:**
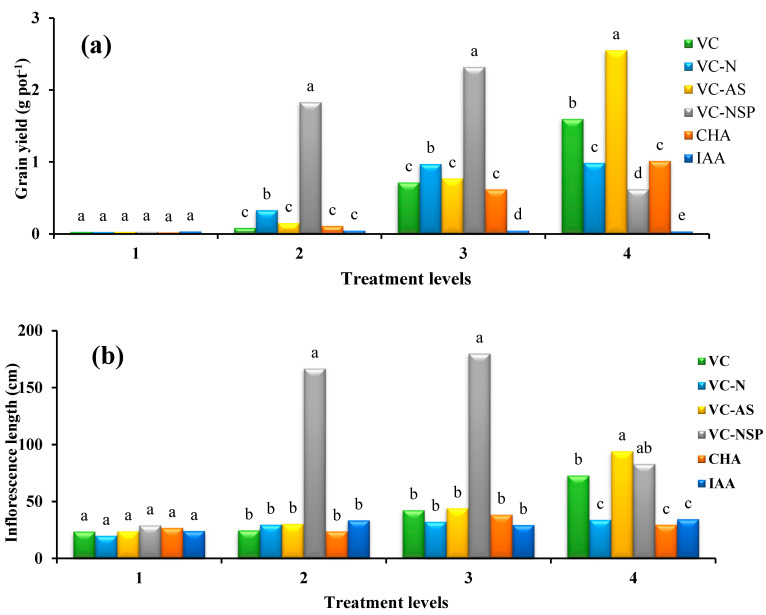
Comparison of average soil application of experimental treatments on Grain yield (**a**) and Inflorescence length (**b**) at treatment levels (levels 1, 2, 3 and 4 for VC, VC-N, VC-AS and VC-NSP treatments of 0, 1, 2 and 3% VC, 0, 200, 400 and 600 mg of CHA per kg of soil and 0, 10^−6^, 10^−5^ and 10^−4^ molar IAA). Different letters in each figure show significant difference at *p ≤* 0.05 by Duncan multiple range test. VC, VC without enrichment; VC-N, VC enriched with 1% nitrogen; VC-NSP, VC enriched with 1% nitrogen, 1% sulfur, and 1% phosphorus; VC-AS, VC enriched with *Azotobacter chroococcum* (21Az) + *Pseudomonas fluorescens* (Ps 59); CHA, Commercial humic acid; IAA, Indole-3-acetic acid.

**Figure 6:**
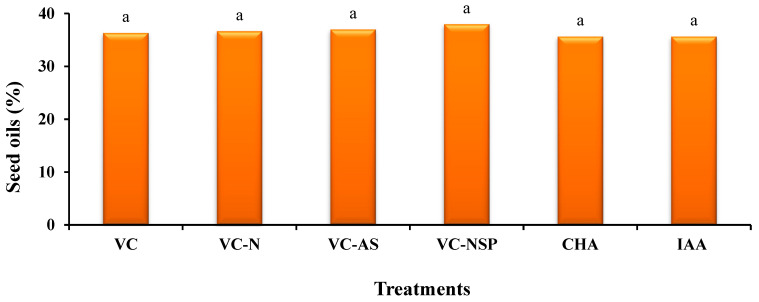
Comparison of average soil application of experimental treatments on seed oils at different treatments. Different letters in each figure show significant difference at *p ≤* 0.05 by Duncan multiple range test. VC, VC without enrichment; VC-N, VC enriched with 1% nitrogen; VC-NSP, VC enriched with 1% nitrogen, 1% sulfur, and 1% phosphorus; VC-AS, VC enriched with *Azotobacter chroococcum* (21Az) + *Pseudomonas fluorescens* (Ps 59); CHA, Commercial humic acid; IAA, Indole-3-acetic acid.

The statistical comparison of mean values showed that the effect of the used treatments as significant at 1% level. Increasing the concentration and percentage of treatments increased the total weight of seeds. Accordingly, VC-AS treatment at 3% level (2.55 g per shrub) had the highest performance while the percentage of effective clusters were increased as well (Figure 5a). Given quasi-hormonal traits and the stimulating impact of HA, increasing the extent of photosynthesis and stomatal conductance in the aforementioned treatments can be the main cause behind the increase in seed weight [76]. Inflorescence length measurement results demonstrated that the longest length belonged to VC-AS treatment (94.2 cm), while the shortest was observed under CHA and IAA treatments. These findings were consistent with the results obtained from the number of clusters and increased number of inflorescence length cluster showed a significant increase as well (Figure 5b). Based on the obtained results, it can be observed that by increasing the load of treatments enriched with N, P, and S, a significant decrease from 3% to 4% can be observed. This decrease could be attributed to the very high sensitivity of plants in the flowering stage, which increased or exceeded nutrients demand in this stage and can have the reverse effect and reduce flowering, growth, and yield [77]. Furthermore, no significant effect was observed between the treatments under measurement in the analysis of oil percentage value (Figure 6). The total amount of oil in rapeseed was reported as 40–45% [78] which was inconsistent with the results of this study. Certain reasons behind such shortage of oil in rapeseed compared to previous reports can be greenhouse cultivation conditions and the lack of fertilizer supply during the growth period. Similarly, Balachandar et al. [51] reported that bacterial-enriched VC had the greatest improvement in plant growth, grain yield, and leaf chlorophyll content. The nutrient content of the plants as well as VC supplemented with C, N, P, and K were significantly increased by enrichment.

### 3.4. Effect of Using Different Treatments on Nutrients Concentrations in the Aerial Organ of Rapeseed

#### 3.4.1. Phosphorus

**Figure 7:**
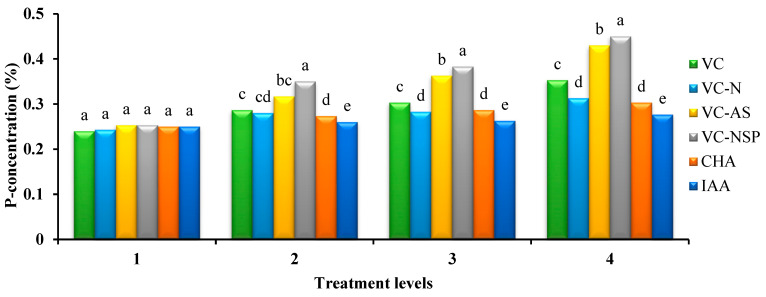
Comparison of average soil application of experimental treatments on P-concentration at treatment levels (levels 1, 2, 3 and 4 for VC, VC-N, VC-AS and VC-NSP treatments of 0, 1, 2 and 3% VC, 0, 200, 400 and 600 mg of CHA per kg of soil and 0, 10^−6^, 10^−5^ and 10^−4^ molar IAA). Different letters in each figure show significant difference at *p ≤* 0.05 by Duncan multiple range test. VC, VC without enrichment; VC-N, VC enriched with 1% nitrogen; VC-NSP, VC enriched with 1% nitrogen, 1% sulfur, and 1% phosphorus; VC-AS, VC enriched with *Azotobacter chroococcum* (21Az) + *Pseudomonas fluorescens* (Ps 59); CHA, Commercial humic acid; IAA, Indole-3-acetic acid.

The results of variance analysis of phosphorus content measured in rapeseed branches at the end of harvest showed a significant statistical difference between various levels of treatments and different treatments. The highest and lowest amounts of phosphorus were obtained from the applications of 3% VC-NSP (0.45%) and indole-3-acetic acid, respectively (Figure 7). Increasing the concentration and consumption percentage of treatments increased the amounts of phosphorus in treatments. Ebrahimi et al. [79] showed that as an organic source, VC enhances access to nutrients including phosphorus, potassium, and iron. Vermicompost enhances phosphorus absorption by increasing phosphorus through activating microorganisms via secreting organic acids such as citric, glutamic, succinic, lactic, oxalic, malic, and fumaric acids or stimulating phosphatase activity [80]. Considering their high phosphorus contents, VC-NSP and VC-AS are probably the causes behind the highest amounts of phosphorus witnessed in the plants placed under these treatments.

#### 3.4.2. Copper

**Figure 8:**
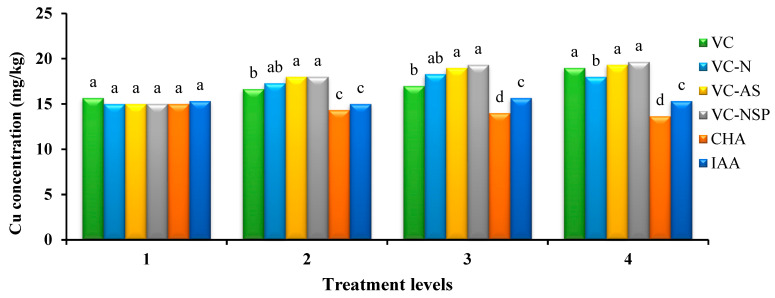
Comparison of average soil application of experimental treatments on P-concentration at treatment levels (levels 1, 2, 3 and 4 for VC, VC-N, VC-AS and VC-NSP treatments of 0, 1, 2 and 3% VC, 0, 200, 400 and 600 mg of CHA per kg of soil and 0, 10^−6^, 10^−5^ and 10^−4^ molar IAA). Different letters in each figure show significant difference at *p ≤* 0.05 by Duncan multiple range test. VC, VC without enrichment; VC-N, VC enriched with 1% nitrogen; VC-NSP, VC enriched with 1% nitrogen, 1% sulfur, and 1% phosphorus; VC-AS, VC enriched with *Azotobacter chroococcum* (21Az) + *Pseudomonas fluorescens* (Ps 59); CHA, Commercial humic acid; IAA, Indole-3-acetic acid.

The results of variance analysis on copper showed a significant difference between the various levels of treatments. The highest and lowest amounts of copper were observed under VC and the CHA treatments (Figure 8). Increasing the concentration and consumption percentage of treatments increased the amounts of copper in treatments. It is reported that HA creates low-soluble complexes with copper, which leads to reduced uptake by plants [81]. Therefore, its content was monitored in canola shoot. Given the measurement of the total copper contents of the plant, the reason for the increased amounts of copper in VC treatments can be attributed to the high extents of growth and dry weights if these treatments. Moreover, the addition of VC bio-fertilizers would enhance access to and absorption of trace elements such as copper by increasing the organic materials of the soil and subsequently, increasing cation exchange capacity [82].

## 4. Conclusions

The role of vermicompost (VC) enrichment treatments is effective in increasing the growth and yield of canola. According to the obtained results, the added of VC at the highest application levels yielded the best result, while the indole-3-acetic acid was found to be the least stimulating amendment that showed the least positive impacts in the measured indices. Vermicomposts NSP (enriched with nitrogen (urea), sulfur, and phosphorus) and AS (bioaugmented with *Azotobacter chroococcum* and *Pseudomonas fluorescens*) treatments involved a significant and higher effect on values of morpho-physiological indices such as height, dry weight of aerial and root organs, SPAD index, and seed performance, compared to other treatments. The VCs NSP and AS treatments involved larger amounts of seeds compared to other treatments. The type of elements which was used for enrichment was very important. The results in this experiment showed that enrichment of VC with urea is the weakest type of enrichment between all elements used. Therefore, for the enrichment of VC, it seems that other enrichment treatments such as ammonium sulfate, etc. should be studied.

## Figures and Tables

**Table 1:** Initial vermicompost properties.

pH	EC (dS·m^−1^)	Total N (%)	OC (%)	P (%)	K (%)	Na (%)	Fe (%)	Ca (%)	C/N
7.63	2.14	1.2	24.37	0.82	6.52	1.1	0.57	8.5	20.3

**Table 2:** Soil amendment composition and application level (1- VC without enrichment (VC); 2- VC enriched with 1% nitrogen (VC-N); 3- VC enriched with 1% nitrogen, 1% sulfur, and 1% phosphorus (VC-NSP); 4- VC enriched with *Azotobacter chroococcum* (21Az) + *Pseudomonas fluorescens* (Ps 59) (VC-AS); 5- Commercial humic acid (CHA); 6- Indole-3-acetic acid (IAA)).

Level 1	Level 2	Level 3	Level 4
No additive of VC	1% VC	2% VC	3% VC
No additive of VC-N	1% VC-N	2% VC-N	3% VC-N
No additive of VC-NSP	1% VC-NSP	2% VC-NSP	3% VC-NSP
No additive of VC-AS	1% VC-AS	2% VC-AS	3% VC-AS
No additive of IAA	10^−6^ molar of IAA	10^−5^ molar of IAA	10^−4^ molar of IAA
No additive of CHA	200 mg kg^−1^ CHA	400 mg kg^−1^ CHA	600 mg kg^−1^ CHA

**Table 3:** Physical and chemical properties of experimental soil.

EC (dS·m^−1)^	pH	Zn (mg kg^−1^)	Fe (mg kg^−1^)	P (mg kg^−1^)	K (mg kg^−1^)	N (mg kg^−1^)	Clay (%)	Sand (%)	Silt (%)
**1.6**	7.2	0.74	2.5	8.1	370	800	33	39	28

## Data Availability

All data, tables, figures and results in paper are our own and original.
